# Impact of six multimodal country-wide campaigns to promote hand hygiene in Belgian hospitals

**DOI:** 10.1186/2047-2994-4-S1-O45

**Published:** 2015-06-16

**Authors:** S Fonguh, N Hammami, B Catry, A Simon

**Affiliations:** 1 Healthcare Associated Infections and Antimicrobial Resistance, Scientific Institute of Public Health Brussels, Brussels, Belgium; 2 Department of Microbiology and Infection control, University Hospital Saint Luc UCL, Brussels, Belgium

## Introduction

Six campaigns sponsored by the Belgian federal government were organized to promote hand hygiene(HH) in Belgian hospitals between 2005 and 2015. The campaigns combined educational sessions for healthcare workers (HCWs), promotion of alcohol-based hand rubs, patient awareness and audits with performance feedback. Each campaign consisted of a pre-campaign data collection period, an awareness period with training and a post-campaign data collection period.

## Objectives

The campaigns aimed at raising awareness on good HH practices and promoting the use of alcohol based hand rubs [1].

## Methods

Using a standardised observation roster, trained infection control teams measured adherence to HH guidelines by direct observation. HH opportunities were counted and the actual episodes of HH were scored as no HH, HH with water and soap, or HH with alcohol-based hand rub. Compliance was stratified by indication and by type of healthcare worker. Compliance was computed as a percentage of the number of episodes divided by the number of opportunities. Each campaign had a specific message geared towards improving compliance, with the message of this year’s campaign being "Hand hygiene, together with the patient".

## Results

Participation rates were excellent for all years, with at least 79% of all hospitals participating voluntarily. National compliance rates increased from 49.6% in 2005 to 69.1% in 2015 before intervention, and from 68.6% in 2005 to 75.8% in 2013 after intervention. After intervention results of 2015 are awaited. Generally nurses performed better than physicians and compliance rates increased for all indications but was always much higher after patient and body fluid contact than before patient contact. Compliance rates increased according to the campaign message for instance physicians compliance increased from 37.6% in 2005 to 53% in 2011 when the campaign message was “Doctors don’t forget, it works and you have a role model”.

## Conclusion

There was a high participation rate in all campaigns. Comparing the effect of all campaigns over time yielded an increase in HH compliance at short and long term when campaigns are repeated regularly, with campaign messages impacting the outcome.

## Disclosure of interest

None declared.
